# Analysis and mapping of a 3^′^ coterminal transcription unit derived from human cytomegalovirus open reading frames UL30–UL32

**DOI:** 10.1186/1743-422X-10-65

**Published:** 2013-02-27

**Authors:** Yanping Ma, Shuang Gao, Lin Wang, Ning Wang, Mali Li, Bo Zheng, Ying Qi, Zhengrong Sun, Weiwei Liu, Qiang Ruan

**Affiliations:** 1 Virus Laboratory, The Affiliated Shengjing Hospital, China Medical University, Shenyang, Liaoning, 110004, China; 2 94K China Medical University, Shenyang, Liaoning, 110004, China

**Keywords:** Human cytomegalovirus, UL30, UL31, UL32, Gene transcription

## Abstract

**Background:**

It has been predicted that the UL31 gene originates from the positive strand of the human cytomegalovirus (HCMV) genome, whereas the UL30 and UL32 genes originate from the complementary strand. Except for the UL32 gene, the transcription of this gene region has not been investigated extensively.

**Methods:**

Northern blotting, cDNA library screening, RACE-PCR,and RT-PCR were used.

**Results:**

At least eight transcripts of the antisense orientation of UL31 were transcribed from the UL30–UL32 region during the late phase of HCMV infection. The 3^′^ coterminus of these transcripts was located within the predicted UL30 gene. The longest 6.0-kb transcript was initiated upstream of the predicted UL32 gene. Other transcripts were derived from the predicted UL30 and UL31 gene region. Except for the previously predicted UL32 open reading frame (ORF), three novel ORFs, named UL31anti-1, UL31anti-2 and UL31anti-3, were located in the transcripts from the UL31anti-UL32 transcription unit. No transcription was found in UL31.

**Conclusion:**

A family of novel 3^′^ coterminal transcripts was transcribed from the UL30–UL32 gene region.

## Background

Human cytomegalovirus (HCMV), a member of the Herpesviridae family, is a ubiquitous pathogen in the human population. HCMV infection in healthy individuals usually displays no signs or has only mild symptoms, and has no long-term health consequences during its life-long latency. Nevertheless, HCMV causes severe congenital abnormalities in neonates and fatal opportunistic infections in immunosuppressed patients [1].

The HCMV genome consists of 230–235 kb of double stranded DNA and is predicted to encode 208 open reading frames (ORFs) in the laboratory strain AD169 [2]. However, 19 additional ORFs, UL133–UL151, have been found in the Toledo strain and other low passaged clinical isolates [3]. Re-evaluation of HCMV coding potential has indicated that 37 previously annotated ORFs ought to be discarded and at least nine previously unrecognized ORFs with relatively strong coding potential should be added [4]. In recent years, several HCMV transcripts have been identified to encode novel ORFs that were not considered in earlier studies [5,6].

It has been predicted that the UL31 gene originates from the positive strand of the HCMV genome, whereas the UL30 and UL32 genes originate from the complementary strand [2]. The initiation site of the UL31 ORF was identified by reanalysis of HCMV Merlin strain genome [7]. The expression of the UL30 and UL32 region has been detected using microarray analysis. However, the orientation of the transcripts has not been identified [8]. Zhang et al. performed the first detailed analysis of antisense transcription in HCMV and identified two groups of transcripts overlapping UL32 and a transcript antisense to UL30 that extended to UL27 [9]. A cDNA clone containing the sequence antisense to the UL31 gene has been found in a late HCMV cDNA library [10].

In the present study, transcription of this gene region was investigated by cDNA screening, northern blotting, RT-PCR and rapid amplification of cDNA ends (RACE). A 3^′^ coterminal transcription unit, named the UL31anti-UL32 transcription unit, was identified in the predicted UL30–UL32 gene region of an HCMV clinical strain. Three novel ORFs were predicted in some of the transcripts from the UL31anti-UL30 transcription unit. No evidence was found for the transcription of the predicted UL31 gene.

## Results

### DNA sequence of UL30–UL32 in HCMV H genome

The whole genome sequence of HCMV H isolate was obtained by high throughput sequencing. The sequence showed that the genome of HCMV H strain contained a completed UL30–UL32 gene region, which shared 99% homology with that of AD169 genome (X17403.1). Compared with AD169 strain, almost all of the nucleotide changes were substitutions, and no frameshift mutation was found in this gene region (Additional file 1: Figure S1).

### UL31 antisense transcripts in HCMV cDNA library

**Figure 1:**
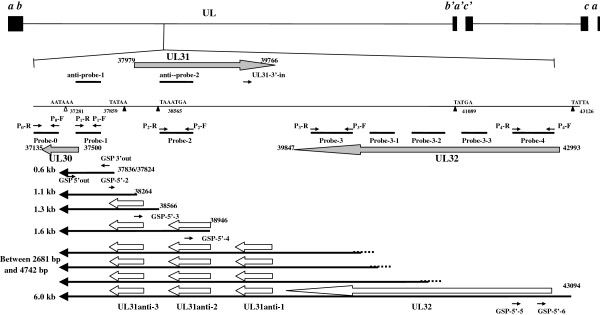
**Graphic representation of the HCMV genome, relative positions of the UL30–UL32 gene region.** The blank arrows represent ORFs; the long black arrows represent transcripts; the dotted lines indicate that the 5^′^ ends were not identified in the present study; the short black arrows represent primers; the black bars represent probes; the black triangles represent TATA elements; the blank triangle represents poly(A) signal. The positions of probes and primers are listed in Table 1. The sizes of transcripts are given at the left sides, and the identified 5^′^ ends are given at the right sides. The nucleotide positions referred to in this figure are in reference to the sequence of the HCMV AD169 strain (GenBank: X17403.1).

Twenty-three cDNA clones were identified to contain sequences congruent with the UL30–UL31 gene region by graded PCR from the library. Compared with the sequences of AD169 strain, the 5^′^ end sequences of 19 clones were located at nucleotide (nt) 37828, three at nt 37824 and one at nt 37836. All of the 23 sequences possessed a poly(A) tail that was not coded by the HCMV genome. The 3^′^ ends of the 23 sequences were all located at nt 37258–37262 downstream of a poly(A) signal (AATAAA) at nt 37281–37276, which was located in the middle of the predicted UL30 gene (Figure 1). The sequencing results of the cDNA clones suggested that an unspliced transcript of ~0.6 kb was transcribed from the UL30–UL31 gene region, which corresponded to the sense orientation of the UL30 gene and antisense orientation of the UL31 gene.

### Confirmation of the UL31anti-UL32 transcription unit by northern blotting

To investigate the sizes of RNA classes from this region of the viral genome, northern blotting was performed using the total RNAs from human embryonic lung fibroblast (HELF) cells infected with HCMV H strain at immediate–early (IE), early (E) and late (L) classes. The total RNA of mock-infected cells was used as a control.

**Figure 2:**
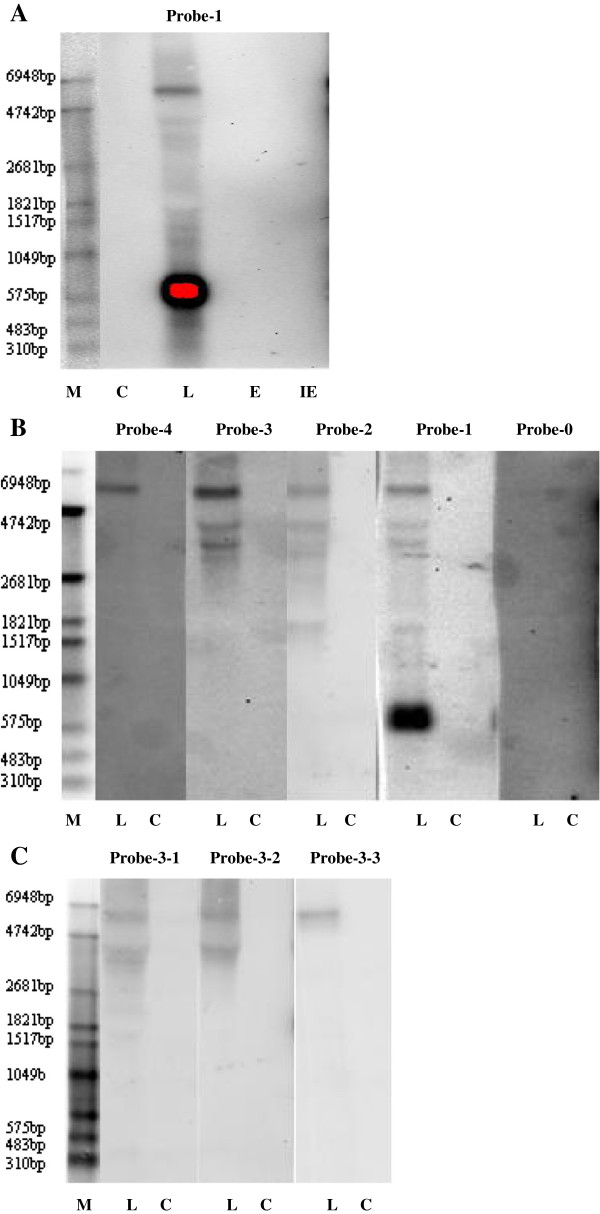
**Northern blotting results of UL31anti-UL32 transcription unit.** The total RNA of mock-infected cells (C) was used as control in all of the experiments. **A**. Northern blottings were performed with Probe-1 using total RNAs harvested from HELF cells infected with HCMV H strain (IE, E, and L classes). **B**. Northern blottings were performed with Probe-0 through Probe-4 using total RNAs harvested from HELF cells at late phase (L) of infection with HCMV H strain. **C**. Northern blottings were performed with Probe-3-1 through Probe-3-3 using total RNAs harvested from HELF cells at late phase (L) of infection with HCMV H strain.

The RNA blots were first hybridized with a digoxigenin-labeled RNA probe, named probe-1, which complemented the complementary strand of the AD169 genome at nt 37365–37664. Multiple transcripts were detected in the L RNA from HCMV-infected HELF cells, but not in the IE, E and mock-infected RNA (Figure 2A). In addition to the most predominant 0.6-kb transcript, a series of larger transcripts was also detected. Among them, one was ~6.0-kb with less abundance than the 0.6-kb transcript, and the other six weak bands were distributed into two groups, lying between the markers of 2681 and 4742 bp, as well as 1049 and 1821 bp, respectively.

To characterize these transcripts further, a series of digoxigenin-labeled RNA probes spanning the UL30–UL32 gene region was designed and used for northern blotting of the RNAs of L-class-infected or mock-infected cells. As shown in Figure 1, Probe-0 was located at nt 37011–37250 downstream of the poly(A) signal within UL30, and contained 116 nt of the 3^′^ terminal sequence of the predicted UL30 ORF. Probe-4 was designed near the 5^′^ end of the predicted UL32 ORF of AD169 strain, which was ~5.0 kb from the poly(A) signal within UL30. Probe-3 and probe-2 were located about 2.7 and 1.4 kb, respectively, from the poly(A) signal. All of the probes bound to the complementary strand of the AD169 sequence (Figure 1, Table 1). As shown in Figure 2B, the 6.0-kb transcript was detected by all of the probes, except for probe-0. In contrast, the transcripts between the markers of 2681 and 4742 bp were detected by probe-1, probe-2 and probe-3, but not probe-4. The longest transcript between the markers of 1049 and 1821 bp was detected by probe-1 and probe-2. The 0.6-kb transcript and the shorter two transcripts between 1049 and 1821 bp were detected only by probe-1. No transcript was detected by probe-0, which suggested that the poly(A) signal at nt 37281–37276 defined the 3^′^ boundary for all of the transcripts. These data indicated that a 3^′^ coterminate transcript family was transcribed from the complementary strand of HCMV UL30–UL32 gene region in the late phase of infection. The region, from which these transcripts were transcribed, was named the UL31anti-UL32 transcription unit.

**Table 1:** The primers used in the present study

**Experiments**	**Primer names**	**Primer sites (5**^′^**-3**^′^**)**	**Primer sequences (5**^**′**^**-3**^**′**^**)**
cDNA library screening	P_1_-F	37664-37645	ACAGCGAGCAGCAGGAGTT
	P_1_-R	37365-37384	AGAGCCCGTCGTGATAGTCC
	P_2_-F	38763-38744	ACCGCCTGCGACTGCCGCAT
	P_2_-R	38564-38583	TACAACAACACGCAGGGCTG
Northern blotting ^*a*^ and RT-PCR	P_0_-F	37250-37231	TAGTCTCGTTTTTTATTAAA
	P_0_-R	37011-37030	CAGTCCGCGACGATCCACAG
	P_1_-F^*b*^	37664-37645	ACAGCGAGCAGCAGGAGTT
	P_1_-R^*b*^	37365-37384	AGAGCCCGTCGTGATAGTCC
	P_2_-F^*b*^	38763-38744	ACCGCCTGCGACTGCCGCAT
	P_2_-R^*b*^	38564-38583	TACAACAACACGCAGGGCTG
	P_3_-F	40306-40288	CGTCGGTGTTCCTTCCTT
	P_3_-R	39997-40014	CATGCCCGTCGTGCTCTT
	P_4_-F	42945-42926	GTCAACTTTCTGCGCCATCT
	P_4_-R	42498-42516	GCTGCACCTCCGTATCCTT
	P_3-1_-F	40673-40654	AGAAACCGGTGCTGGGCAAG
	P_3-1_-R	40430-40447	ACGGACGCCGAGGCTGAC
	P_3-2_-F	41023-41005	TCCCTTCAGGATGCCTACG
	P_3-2_-R	40784-40801	TCGGACGACGGTGTTGTG
	P_3-3_-F	41361-41344	GATCCGCGTTTCACCGAC
	P_3-3_-R	41115-41132	CCCAGGGCGAGTTACCGT
3^′^ RACE	GSP-3’out primer	37713- 37694	CATGTAGCCGACTTGGAGGA
	P_1_-F	37664-37645	ACAGCGAGCAGCAGGAGTT
	P_2_-R	38564 -38583	TACAACAACACGCAGGGCTG
	UL31-3’-in	39310-39327	CCGCAACCCGTCACTCTT
5^′^ RACE	GSP-5’out primer	37342- 37359	AAAGGCACGCTGTTGACG
	P_1_-R	37365-37384	AGAGCCCGTCGTGATAGTCC
	GSP-5’-2	37801-37820	CGGGAAGAGGTTCTTCTCCC
	GSP-5’-3	38349-38367	ACGTGGTGACCTCGTGGAT
	GSP-5’-4	38743-38762	CATGCGGCAGTCGCAGGCGG
	GSP-5’-5	42522-42539	GCACAAAGGCGATGGGTT
	GSP-5’-6	42803-42823	CGGTAGTATCCCAACCAAAGC

*a*. The additional oligonucleotide of 5^′^-AATACGACTCACTATAGG-3^′^ was added to the 5^′^ ends of the reverse primers for the templates of all the Northern blotting probes and acted as the promoter of T7 RNA polymeras.

*b*. These primers were for the template of the complementary probes of probe-1 and anti-probe-1, as well as probe-2 and anti-probe-2, which were switched as the forward and reverse primers.

To distinguish further the three transcripts between the markers of 2681 and 4742 bp, another three digoxigenin-labeled RNA probes, probe-3-1, probe-3-2 and probe-3-3, were designed lying at the right side of probe-3 in order, and downstream of (probe-3-1 and probe-3-2) or upstream of (probe-3-3) a non-consensus TATA element (TATGA) at nt 41089–41084 (Figure 1, Table 1). The probes were hybridized to the RNAs from L-class-infected cells or mock-infected cells. The 6.0-kb transcript was detected by all three probes. The three transcripts between the markers of 2681 and 4742 bp were recognized by probe-3-1. Among them, the shortest one was detected with a weak signal, which could have been due to hybridization to the partial sequence of the probe. Only the longest transcript reacted to probe-3-2, and none of the three transcripts was detected by probe-3-3. No signal was found in mock-infected RNA using all three probes (Figure 2C). These results demonstrated that all three transcripts should initiate between the positions of probe-3-1 and probe-3-3, and downstream of the TATA element (TATGA) at nt 41089–41084.

### Identification of the 3^′^ terminus of the transcripts from the UL31anti-UL32 transcription unit by 3^′^ RACE

**Figure 3:**
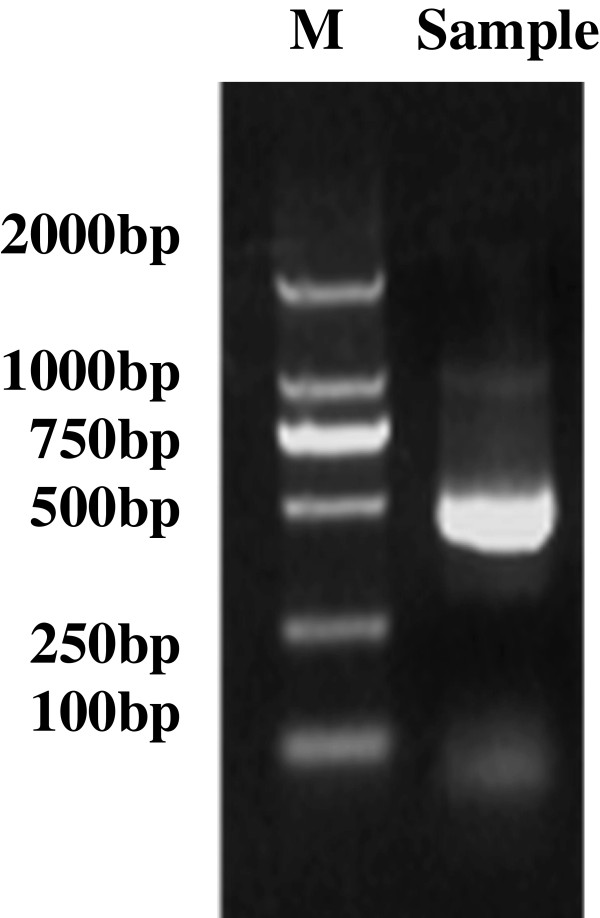
**3**^′^**RACE result of transcripts from the UL31anti-UL32 transcription unit using the L class RNA of HCMV infected HELF cells as the sample.**

To identify the 3^′^ end of these 3^′^ coterminate transcripts by 3^′^ RACE, gene-specific primers (GSP) of GSP-3^′^ out primer and P_1_-F primer (Figure 1, Table 1) were used in the amplification. The two nested primers were located in the region of the 0.6-kb transcript, which is derived from the 3^′^ part of the UL31anti-UL32 transcription unit. The cDNA was reverse transcribed from L class RNA of infected cells. The product of 3^′^ RACE was ~400 bp (Figure 3). Sequencing demonstrated that the 3^′^ end of the transcripts from the UL31anti-UL32 transcription unit was located at nt 37260, which was downstream of the consensus poly(A) signal at nt 37281–37276. These results were identical to those of the transcripts identified by screening the cDNA library and northern blotting.

### Detection of possible introns in the transcripts from the UL31anti-UL32 transcription unit by RT-PCR

**Figure 4:**
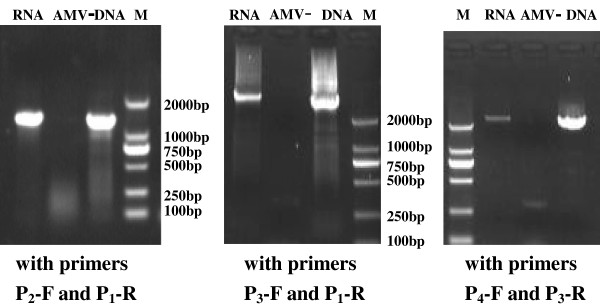
**RT-PCR results of the transcripts from the UL31anti-ul32 transcription unit.** The reactions with DNA as the template and omitting AMV reverse transcriptase were performed as controls.

To detect possible introns in the transcripts from the UL31anti-UL32 transcription unit, RT-PCR was performed using L class RNA of infected cells as the template. Total RNA was subjected to RT-PCR with primer combinations of P_2_-F and P_1_-R, P_3_-F and P_1_-R, as well as P_4_-F and P_3_-R, respectively (Figure 1, Table 1). Control reactions containing no avian myoblastosis virus (AMV) reverse transcriptase were performed. Meanwhile, PCR using the infected-cell DNA as template and the same primers in parallel tests were used as a length control. The results showed that the lengths of RT-PCR products were identical to those of the PCR products with DNA as the template using the same primers. No fragment in the AMV-negative control reactions was amplified, which excluded possible DNA contamination in the RNA preparations (Figure 4). This result indicated that no intron existed in the transcripts from the UL31anti-UL32 transcription unit.

### Identification of the 5^′^ termini of the 0.6- and 6.0-kb transcripts by 5^′^ RACE

**Figure 5:**
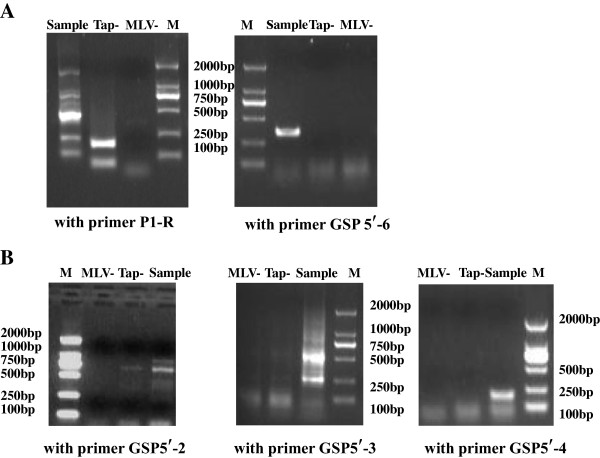
**5**^**′**^**RACE results of the transcripts from the UL31anti-UL32 transcription Unit using the L class RNA of HCMV infected HELF cells as the sample. A**. 5^′^ RACE results of the 0.6 and 6.0 kb transcripts using primers of P1-R and GSP5^′^-6 as the gene specific 5^′^ RACE primer, respectively. All the controls of TAP (−) and M-MLV (−) were negative, except for the TAP (−) control when amplified with primer P1-R. The product of the TAP (−) control was not consistent with those of the sample, and was not sequenced. **B**. The 5^′^ RACE results of the transcripts between 1049 and 1821 bp using primers of GSP5^′^-2, GSP5^′^-3 and GSP5^′^-4 as the gene specific primer, respectively. After amplification with primer GSP 5^′^-2, a product similar to the 750 bp product of the sample was found in the TAP (−) control. However, sequencing result showed that the 5^′^ end was not consistent with that of the 750 bp product of the sample.

5^′^ RACE experiments were performed using primers designed according to the 0.6- and 6.0-kb transcripts. For the 0.6-kb transcript, the first round of PCRs was carried out using the 5^′^ RACE outer primer (from the 5^′^ Full Race Kit) and the GSP-5^′^ outer primer (Table 1, Figure 1). Several bands were obtained using P_1_-R as the inner primer (Figure 5A). A predominant band of ~300 bp was recovered, cloned and sequenced. The sequences of all the selected clones initiated at nt 37836 or 37824, which were consistent with the 5^′^ ends of the cDNA sequences identified by cDNA library screening. A TATA box (TATAA) was found at nt 37865–37859, which was located 30 bp upstream of the 5^′^ ends of the 0.6-kb transcript (Additional file 1: Figure S1).

After amplification with the GSP-5^′^-5 and GSP-5’-6 primers, an ~300-bp product was purified, cloned and sequenced. The sequencing results of all the selected clones indicated an accordant 5^′^ end at nt 43094, which was 27 bp downstream of a TATA element at nt 43126–43121 (TATTA) (Additional file 1: Figure S1). Combined with the results obtained in 3^′^ RACE and RT-PCR, an unspliced transcript could be deduced to initiate at this site and terminate at the 3^′^ coterminus of nt 37260. After adding one 100–200-bp poly(A) tail, this transcript was coincident with the 6.0-kb transcript identified by northern blotting.

### Identification of the 5^′^ termini of the transcripts with lengths of 1049–1821 and 2681–4742 bp by 5^′^ RACE

To define the 5^′^ ends of the transcripts with lengths of 1049–1821 bp, several nested 5^′^ RACE PCR experiments were performed. The first-round PCR was carried out using the 5^′^ RACE Outer primer (from the 5^′^ Full Race Kit) and the P_1_-R primer (Table 1, Figure 1). The nested amplification results were as follows: three bands of ~900, ~750 and ~400 bp were obtained using GSP-5^′^-2 primer; two bands of ~500 and ~250 bp using the GSP-5^′^-3 primer; and one band of ~200 bp using the GSP-5^′^-4 primer. These products were purified and cloned separately. Three to six clones of each product were sequenced for the inserts. As shown in Table 2, the sequencing results indicated three possible 5^′^ ends around nt 38946, 38566 and 38264, respectively. So, the three transcripts located between 1049 and 1821 bp in the northern blot results should be 1.6, 1.3 and 1.1 kb in length before polyadenylation.

**Table 2:** **The detailed 5**^′^**RACE results of the transcripts between 1049 bp and 1821 bp**

**GSP 5**^′^**Inner primers**	**Primer sites**	**Products**	**5**^′^**ends**	**The number of clones**
GSP-5^′^-2	37801-37820	900 bp	38724	3
		750 bp	38558	1
			38568	1
			**38566**	4
		400 bp	38267	1
			**38264**	5
GSP-5^′^-3	38349-38367	500 bp	**38946**	1
			38830	3
		250 bp	**38566**	4
			38262	1
GSP-5^′^-4	38743-38762	200 bp	**38946**	3
			38950	1

The consistent 5^′^ ends were in bold type.

According to the UL30–UL32 DNA sequence, the prediction of transcription regulation motifs showed that except for a non-consensus TATA element (TAAATGA) at the corresponding position of nt 38565–38559 with AD169 as the reference (Additional file 1: Figure S1), no consensus TATA element was found upstream of the corresponding gene positions of the 5^′^ ends of the three transcripts. However, several other transcription-activating sequences were observed upstream of the corresponding gene positions of these 5^′^ ends, including an E2F site (TTACGCGC) and a CREB/CEBP site (CGTGGTTACGCG) at 38 and 41 bp relative to the corresponding gene position of the 5^′^ end of nt 38946, respectively; a GATA site (AGGGATACCG) at 10 bp relative to the 5^′^ end of nt 38264 (Additional file 1: Figure S1).

The same strategy was applied to define the 5^′^ ends of the transcripts of 2681–4742 bp. Despite the use of multiple nested 5^′^ RACE primers, the results did not provide any authentic identical 5^′^ ends. DNA statistics and RNA fold analysis on the corresponding genomic sequence showed that high GC percentage (63.72%) and complicated secondary structures (hairpins or stem-loop caps) existed in this region, which may have blocked the reverse transcription. Transcription regulation site analysis showed that there were three Cap sites (TCACCTGG, TCAGCCTG and TCAGCCTC) at the positions corresponding to AD169 genome nt 41038–41031, 40823–40816 and 40446–40439, respectively. A non-consensus TATA element (TATGA) was located at nt 41089–41084, which should be upstream of the longest transcript of 2681–4742 bp. In addition, two AP-1 sites at nt 40615–40605 (GGTGACGTCGAC) and 40939–40929 (GGTGACGTCGA), a CREB/ATF site at nt 40616–40604 (CGGTGACGTCGACG), and an E2F site at nt 40489–40481 (TTCTGGCGC) were found in the corresponding genomic sequence (Additional file 1: Figure S1).

### Detection of transcription of the predicted UL31 gene by northern blotting

Transcription of the predicted UL31 gene was detected by two digoxigenin-labeled RNA probes, named anti-probe-1 and anti-probe-2, which were complementary to the sequences of probe-1 and probe-2, respectively (Figure 1). Northern blotting was performed using the total RNAs from HELF cells infected with HCMV H strain at IE, E and L classes. No transcript was detected by either of the probes in all the RNA preparations of the three infection classes (data not shown).

### Detection of transcription of the predicted UL31 gene of by 3^′^ RACE

To determine whether the predicted UL31 gene was transcribed, 3^′^ RACE was performed with GSPs of P_2_-R and UL31-3^′^-in (Figure 1, Table 1) using L class RNA of infected cells. No product was found after nest amplification.

### Coding potential prediction of the transcripts from the UL31anti-UL32 transcription unit

The potential ORFs in transcripts from the UL31anti-UL32 transcript unit were analyzed with ATG as the initiation codon. In addition to the UL32 ORF (nt 42993–39847), three novel ORFs were predicted, named UL31anti-1, UL31anti-2 and UL31anti-3, which spanned nt 39760–39320, 38938–38375 and 38313–37903, respectively, with the AD169 genome as the reference (Additional file 1: Figure S1). The ORFs were predicted to encode 147, 188 and 137 amino acids, respectively. As shown in Figure 1, the 6.0-kb transcript comprised all of the four ORFs, and the three transcripts of 2681–4742 bp comprised the three newly predicted ORFs. The 1.6-kb transcript contained UL31anti-2 and UL31anti-3 ORFs, whereas the 1.3-kb transcript only encoded the UL31anti-1 ORF. No coding potential was predicted in the 0.6- and 1.1-kb transcripts (Figure 1).

## Discussion

Evidence of transcription of the UL32 gene has been obtained previously [11-13]. Recently, a 0.55-kb transcript has been proved to be transcribed from the complementary strand of HCMV Merlin strain in the UL30–UL31 gene region [13]. In the present study, a transcription unit at the complementary strand of this gene region was identified in an HCMV clinical strain, and named the UL31anti-UL32 transcription unit. A family of 3^′^ coterminal transcripts was transcribed during the late phase of HCMV infection, whose 3^′^ terminus was located within the middle of the predicted UL30 gene. The transcript family included at least eight mRNAs, of which the 6.0-kb transcript was consistent with that of the previously identified UL32 transcript, which has been shown to encode the UL32 gene product (pp150) [11], and the 0.6-kb transcript accords with the 0.55-kb transcript reported by Gatherer et al. [13]. The other six were novel transcripts.

In addition to UL32, three novel ORFs, UL31anti-1, UL31anti-2 and UL31anti-3, were predicted in six transcripts from the transcription unit. These ORFs were located within the UL31 gene region but had an antisense orientation. Translation of small ORFs from the UL30–UL32 region had been documented recently [14]. It would be worthwhile investigating the expression of these three novel ORFs in the future.

No ORF was found in the shortest two transcripts (0.6 and 1.1 kb), when predicted with ATG as the initiation codon. However, a comparative analysis involving members of the primate cytomegalovirus (HHV5, PnHV2, CeHV5, CeHV8, AoHV1 and SaHV3) led to the discovery of a potential coding region (UL30A) located in the 0.55-kb transcript, potentially expressed from a nonconventional initiation codon of ACG [15]. Thus, the coding potential of the 0.6- and 1.1-kb transcripts should be investigated further at the protein level.

In the present study, northern blotting using probe-0, which binds to the 3^′^ end of the predicted UL30 sequence, did not detect any transcript. Furthermore, the transcription evidence of the UL31 gene has not been obtained by 3^′^ RACE and northern blotting. These results suggest that the previously predicted UL30 and UL31 genes should not be transcribed.

On the basis of the presence of consensus TATA elements located 15 or 30 bases upstream of the mapped initiation sites in the genomic sequence, the 0.6- and 6.0-kb transcripts appear to initiate from a TATA-containing promoter. No consensus TATA element was found in the upstream sequences of the two groups of weak transcripts of 2681–4742 and 1049–1821 bp. Northern blotting showed that the transcription activity of the sequences upstream of the two groups of weak transcripts was weaker than that of the consensus TATA elements upstream of the 0.6- and 6.0-kb transcripts. Several other transcription-activating sequences were observed in the upstream sequences of the two group transcripts, including the non-consensus TATA element, AP-1 site, CREB/ATF site, and E2F site. Although all of these elements have been identified in other HCMV transcription units [16-18], their regulatory activity for this transcription unit should be determined in future research.

Although the expression of 151 predicted HCMV ORFs has been detected by DNA microarray analysis [12], the transcription characteristics of nearly half of the predicted genes have not been investigated extensively. More unrecognized translated products or transcripts could be involved in HCMV pathogenesis. Detailed mapping of HCMV transcripts may provide much more important information for understanding the interactions between HCMV and its hosts. Further investigation on the novel transcripts may offer new strategies for intervention and treatments of HCMV-related diseases.

## Conclusions

In this study, a family of 3^′^ coterminal transcripts was transcribed in the late phase of HCMV infection from the predicted UL30–UL32 gene region of a clinical strain.

## Methods

### Virus and specimens

The HCMV clinical strain H was isolated from a urine sample of an infant aged <5 months who had been hospitalized in Shengjing Hospital of China Medical University. The strain was passaged 10 times in HELF cells, which were maintained in 1640 medium supplemented with 2% fetal calf serum and 100 U penicillin/streptomycin at 37°C and 5% CO_2_ in a humidified incubator. HELF cells were inoculated with H strain at an MOI of 3–5.

### DNA sequencing of HCMV H strain

The genome of HCMV H strain was sequenced by high-throughput sequencing, using a Genome Sequencer Illumina Hiseq2000 (Huada, Shenzhen, China).

### RNA preparations

For preparation of HCMV IE RNA, 100 μl/ml protein synthesis inhibitor cycloheximide (Sigma, St Louis, MO, USA) was added to the culture medium 1 h before infection, and the cells were harvested at 24 h post-infection (hpi). For E RNA preparation, 100 μl/ml DNA synthesis inhibitor phosphonoacetic acid (Sigma) was added to the medium immediately after infection, and the cells were harvested at 48 hpi. HCMV L RNA and mock-infected cellular RNA were derived from infected and uninfected cells, respectively, cultured in parallel, and harvested at 96 hpi. Total RNA was isolated from ~10^7^ infected or uninfected HELF cells using TRIzol reagent (Invitrogen, Carlsbad, CA, USA). The isolated RNA was treated with DNA-free reagent (Ambion, Austin, TX, USA) to remove possible contaminating DNA. The integrity and size of the isolated RNA was analyzed by formaldehyde agarose gel electrophoresis. The quantity and purity of the RNA were estimated by OD measurement.

### Screening an HCMV cDNA library

An HCMV cDNA library had been constructed previously using the Switching Mechanism At 5^′^ end of RNA Transcript (SMART) technique (Clontech, Mountain View, CA, USA) and the L RNA of HCMV H strain [10]. To select specific cDNA clones from the cDNA library by PCR, a graded PCR was set up as previously described [19,20]. Five thousand cDNA clones were screened by graded PCR using several pairs of primers (Table 1, Figure 1). The PCR conditions were: initial denaturation of templates at 94°C for 4 min, 30 cycles of 94°C for 30 s, 55°C for 30 s, and 72°C for 1 min, followed by a final elongation at 72°C for 10 min. Inserts of the selected clones were sequenced using vector primers (M13F and M13R) and the ABI PRISM 3730 DNA analyzer (Applied Biosystems, Carlsbad, CA, USA). The screening results allowed us to obtain cDNA clones containing transcript sequences derived from both genomic strands.

### Northern blotting

For northern blotting analysis, 10 μg per lane of IE, E and L RNA preparations of the HCMV H strain and total RNA from mock-infected HELF cells were subjected to denaturing agarose gel (1% w/v) electrophoresis in the presence of formaldehyde, alongside the digoxigenin-labeled RNA molecular weight marker I (Roche, Indianapolis, IN, USA). Probes were labeled using a DIG Northern starter kit (Roche) according to the manufacturer’s instructions. Primers for producing the probes are listed in Table 1 and Figure 1. The separated RNA fragments were transferred onto positively charged nylon membranes using capillary transfer. The nylon membranes were baked at 80°C for 2 h followed by prehybridization for 30 min at 65°C using the Dig EasyHyb-buffer (Roche). After overnight hybridization to the probes at 65°C, the membranes were washed according to the manufacturer’s instructions. The hybridized probes were incubated with anti-digoxigenin conjugated to alkaline phosphatase and visualized with the chemiluminescence substrate CDP-Star (Roche). The membranes were exposed using ChemiDocTM XRS + (Bio-Rad, USA). To ensure that equal amounts of RNA were loaded, the RNA preparations were adjusted by comparing with the quantities of 28S and 18S rRNA in the same RNA preparations, estimated by electrophoresis and ethidium bromide staining.

### RACE

3^′^ and 5^′^ RACE was performed with 3^′^-Full RACE Core Set Ver. 2.0 and 5^′^-Full RACE Kit (TaKaRa, Dalian, China), respectively. L RNA preparations from the H strain were used as templates. First-strand cDNAs were synthesized with Moloney murine leukemia virus (MMLV) reverse transcriptase using oligo-dT-adaptor primers (3^′^ RACE) and random 9-mer primers (5^′^ RACE), respectively. Nested PCR amplifications were carried out using LA Taq (TaKaRa) after reverse transcription. All of the primers are listed in Table 1 and Figure 1. The reactions were carried out at 94°C for 4 min, 30 cycles of 94°C for 30 s, 55–58°C (depending on primers) for 30 s, and 72°C for 30 s to 5 min, with a final extension at 72°C for 10 min. In 5^′^ RACE experiments, two control reactions were performed in strict accordance with kit instructions: (i) TAP(−), omitting tobacco acid pyrophosphorylase, to rule out nonspecific ligation of incomplete mRNA, tRNA and rRNA with the adaptor; and (ii) MMLV(−), omitting MMLV reverse transcriptase, to rule out nonspecific reaction caused by contaminating genomic DNA.

### RT-PCR

RT-PCR was performed using L RNA preparations from the H strain. First-strand cDNA was synthesized using Oligo-dT adaptor primer and AMV reverse transcriptase (TaKaRa). Gene-specific cDNA sequences were amplified using appropriate GSP combinations of P_2_-F and P_1_-R, P_3_- F and P_1_-R, as well as P_4_-F and P_3_-R (Figure 1, Table 1).

### Cloning and sequencing

Products of RACE and RT-PCR were separated by agarose gel electrophoresis and purified using the DNA Purification Kit (Promega, Madison, WI, USA). Recovered PCR products were ligated into a pCR 2.1 TA vector (Invitrogen, China) with T4 ligase at 14°C overnight. The ligation products were transformed into *Escherichia coli* DH/5α competent cells. Three to six clones of each purified PCR product were selected randomly for sequencing using the M13 primer and the ABI PRISM 3730 DNA analyzer (Applied Biosystems).

### BLAST search and sequence analysis

Standard nucleotide–nucleotide BLAST was performed on the NCBI website. The nucleotide positions referred to in this study were in reference to the sequence of the HCMV AD169 strain (GenBank: X17403.1). DNA alignment was done by MegAlign using Clustal W algorithms. RNA fold analysis and ORFs prediction were performed by GeneQuest and Editseq programs of the DNAstar package. The prediction of transcription regulation motifs was performed using Searching Transcription Factor Binding Sites (Ver. 1.3, Japan) on line.

## Abbreviations

HCMV: Human cytomegalovirus; ORF: Open reading frame; HELF: Human embryonic lung fibroblast; IE: Immediate early; E: Early; L: Late; RACE: Rapid amplification of cDNA ends; SMART: Switching Mechanism At 5^′^ end of RNA Transcript

## Competing interests

The authors declare that they have no competing interests.

## Authors’ contributions

YPM carried out primer design, PCR and sequence analysis. As the corresponding author, QR conceived the experimental design and participated in revising the manuscript. NW and MLL carried out virus preparation and cell culture. BZ and SG carried out Northern blot analysis. LW and YQ carried out plasmid construction. ZRS and WWL carried out RNA isolation. All authors have read and approved the final manuscript.

## Supplementary Material

Additional file 1: Figure S1 Alignment of UL30-UL32 DNA sequences of HCMV H strain and AD169 strain. Relative positions are shown as AD169 strain (GenBank: X17403.1). The predicted ORFs are denoted by square brackets. The 5^′^ends of the transcripts are remarked by triangles. The regulate elements are remarked by barres. Click here for file
