# Supplementary material for: Analysis and mapping of a 3′ coterminal transcription unit derived from human cytomegalovirus open reading frames UL30–UL32
Source: Virol J. 2013 Feb 27;10:65. doi: 10.1186/1743-422X-10-65 (PMC3600006; doi:10.1186/1743-422X-10-65)
Supplement: Additional file 1: Figure S1 — Alignment of UL30-UL32 DNA sequences of HCMV H strain and AD169 strain. Relative positions are shown as AD169 strain (GenBank: X17403.1). The predicted ORFs are denoted by square brackets. The 5′ends of the transcripts are remarked by triangles. The regulate elements are remarked by barres. [file 1743-422X-10-65-S1.doc]

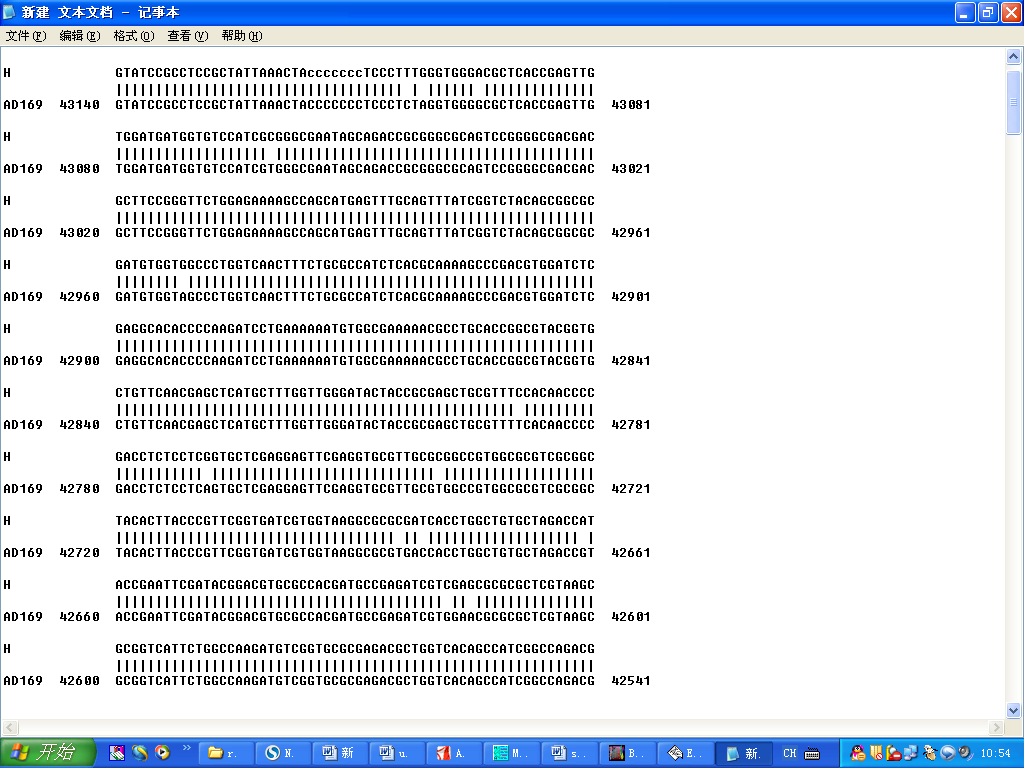


**TATA, nt43126-4312**1

**5’end, nt43094**

**UL32 ORF, nt429934**


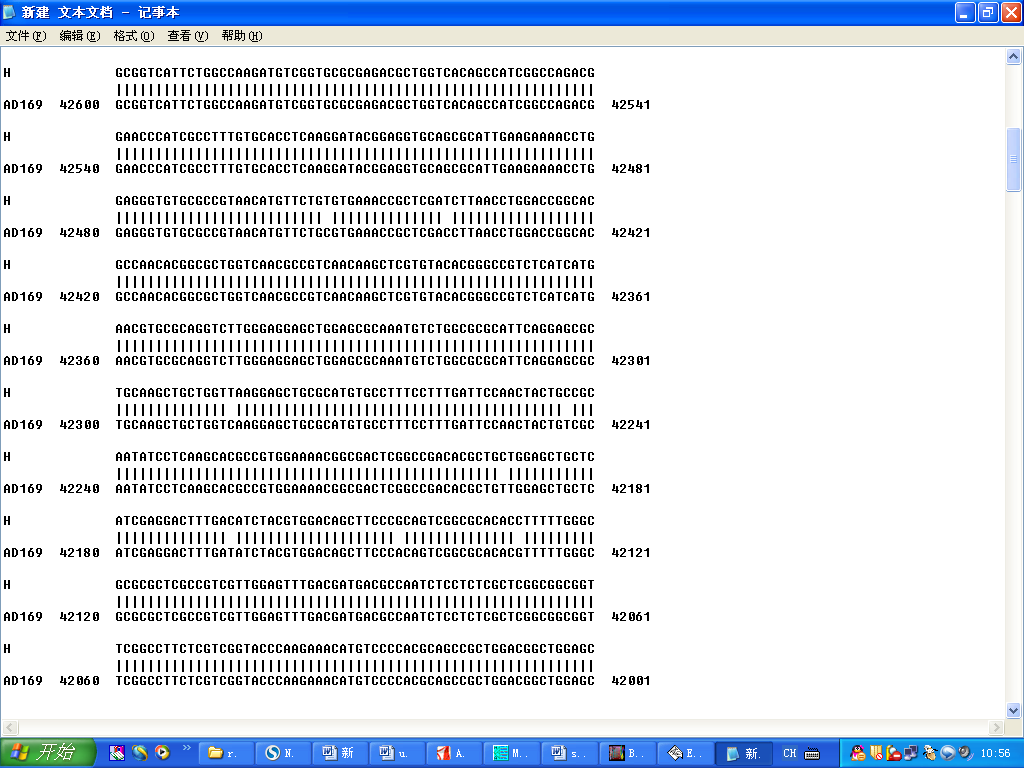


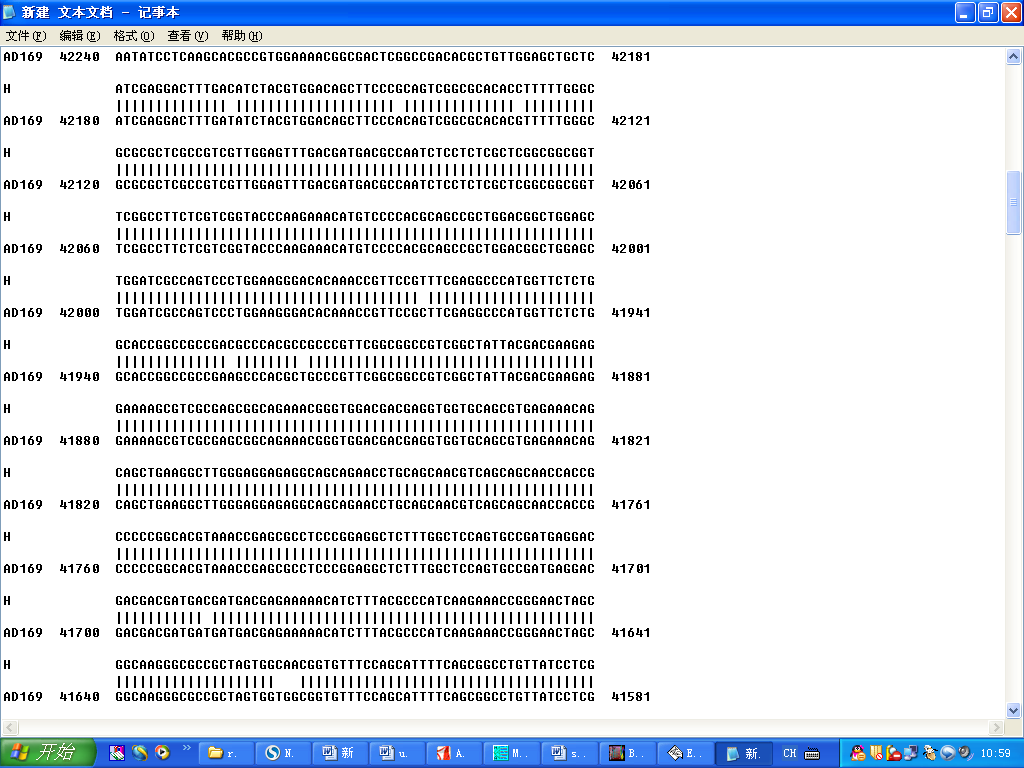


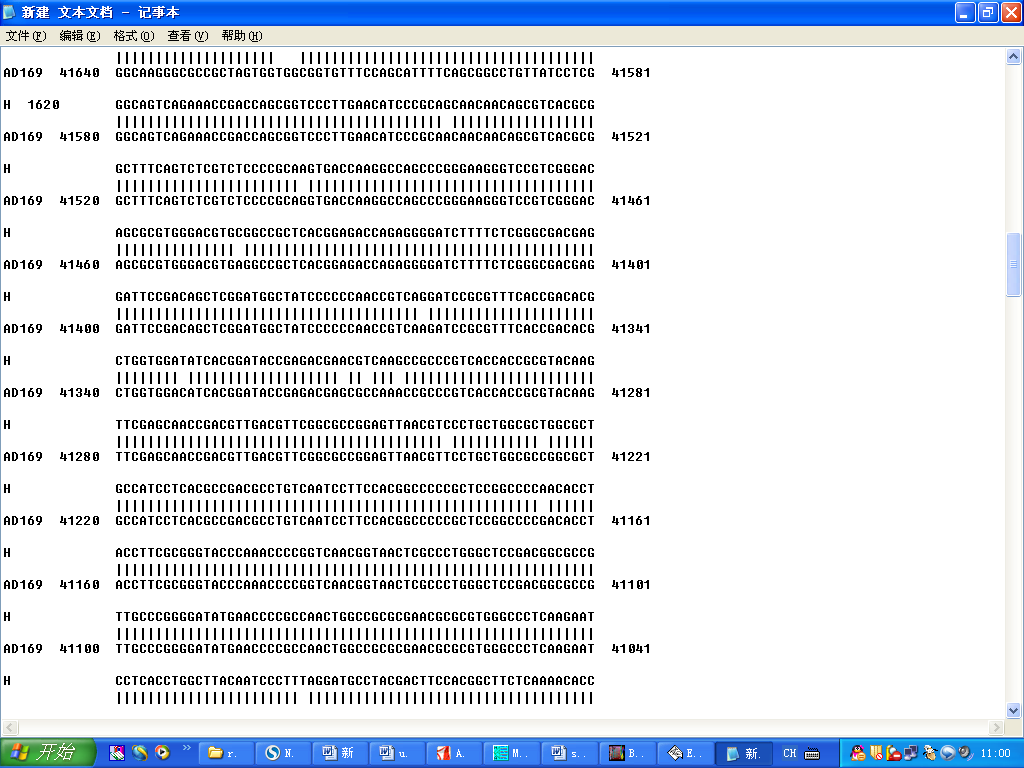


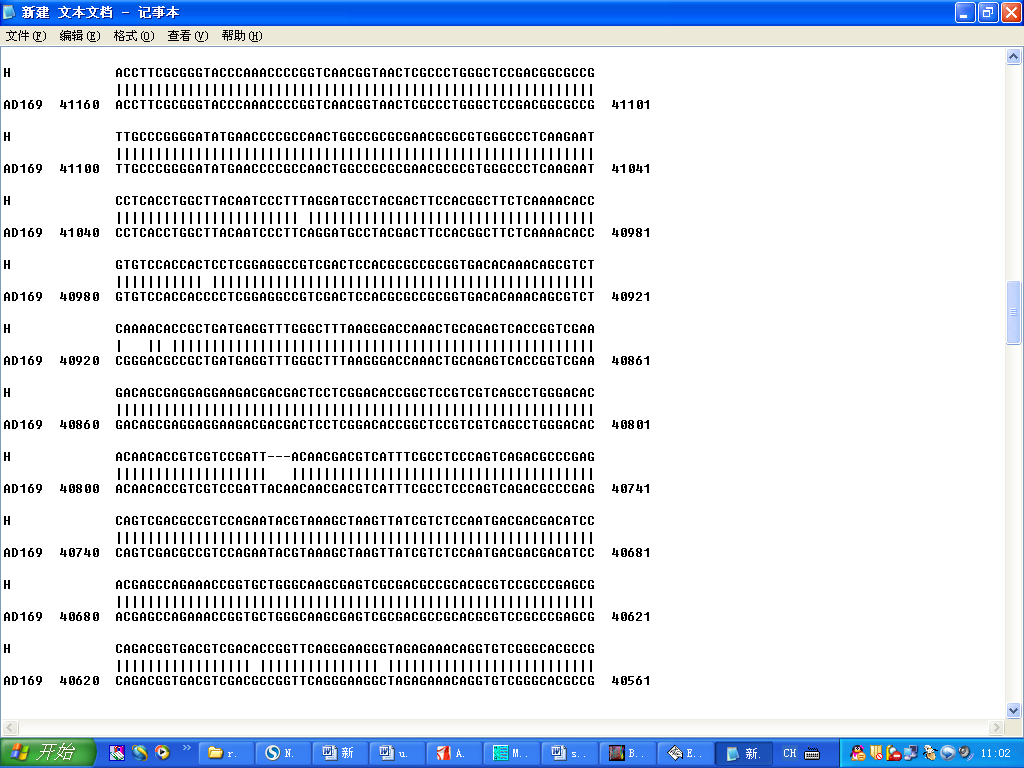


**Cap, nt 41038-41031**

**Ap-1, nt 40939-40929**

**Cap, nt40823-40816**

**AP-1/CREB/ATG,, nt40616-40605**


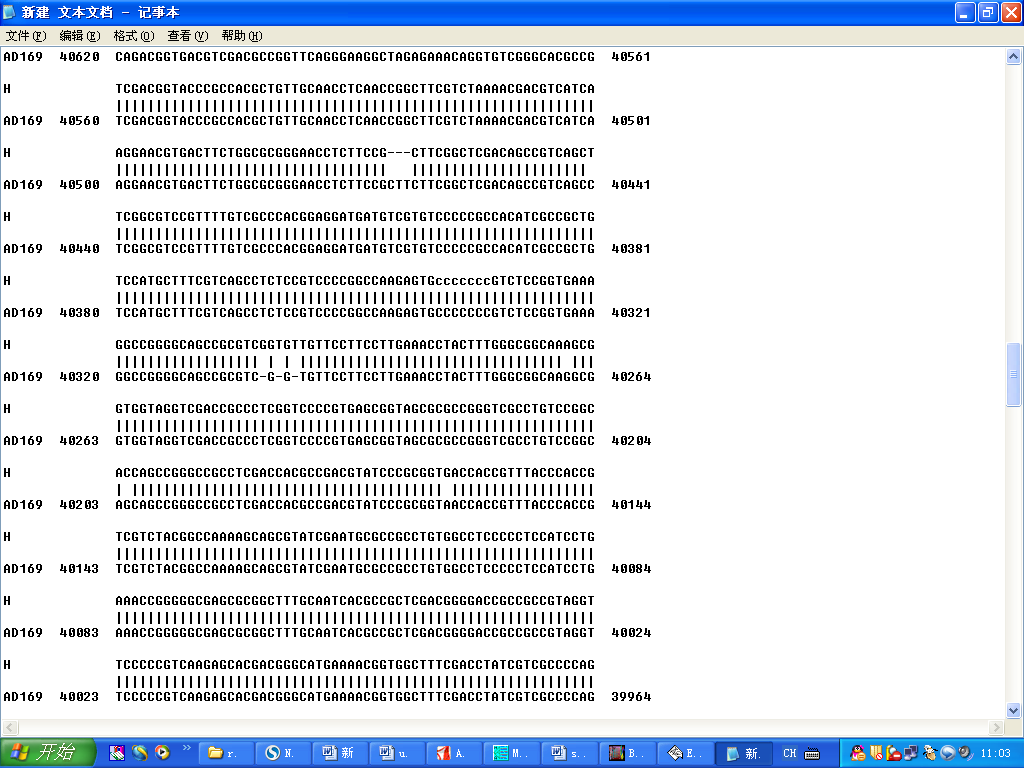


**E2F, nt 40489-40481**

**Cap, nt40446-40439**


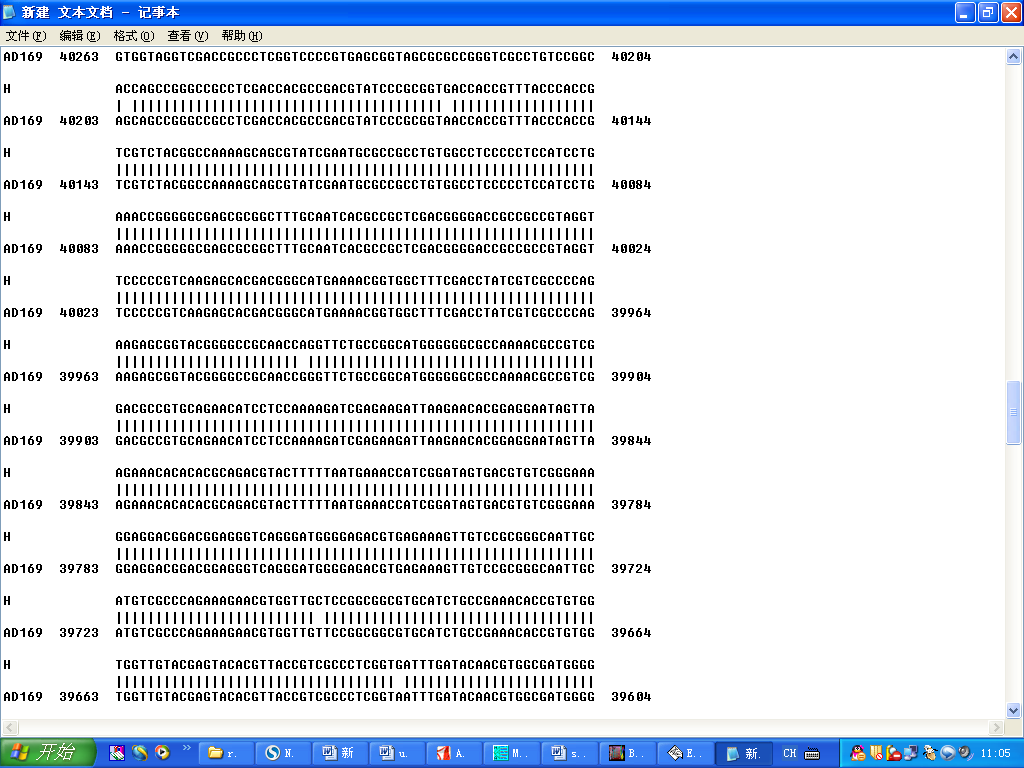


**UL32 ORF, nt 39847**

**UL31anti-1 ORF, nt 39760**


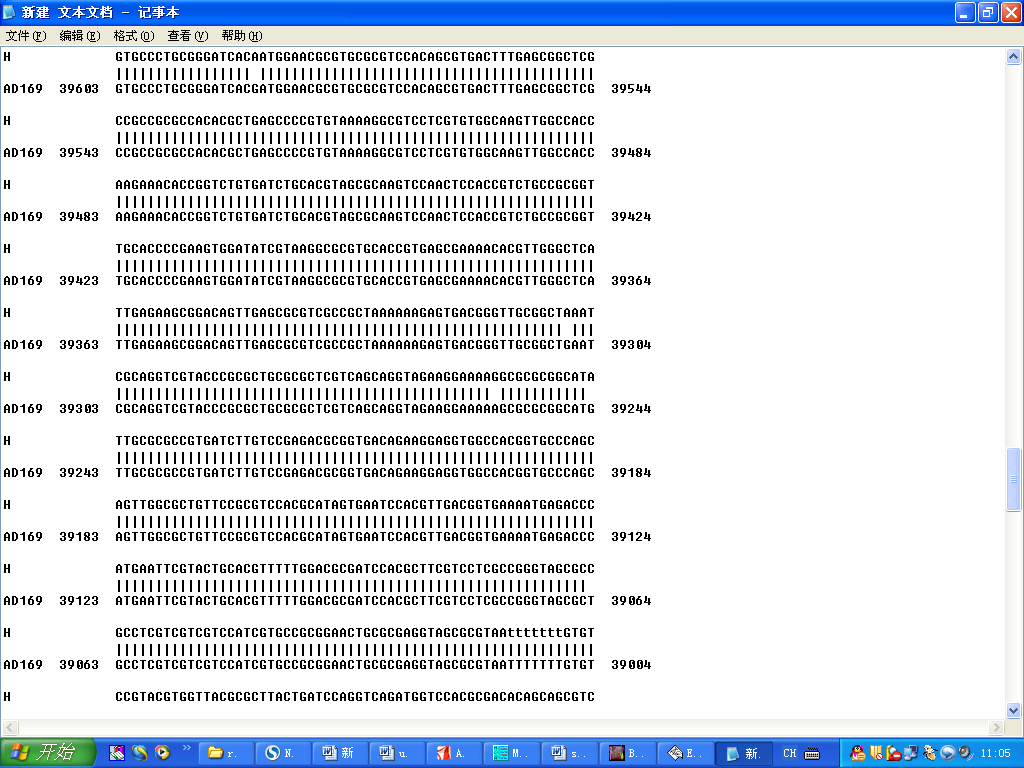


**UL31anti-1 ORF, nt 39320**


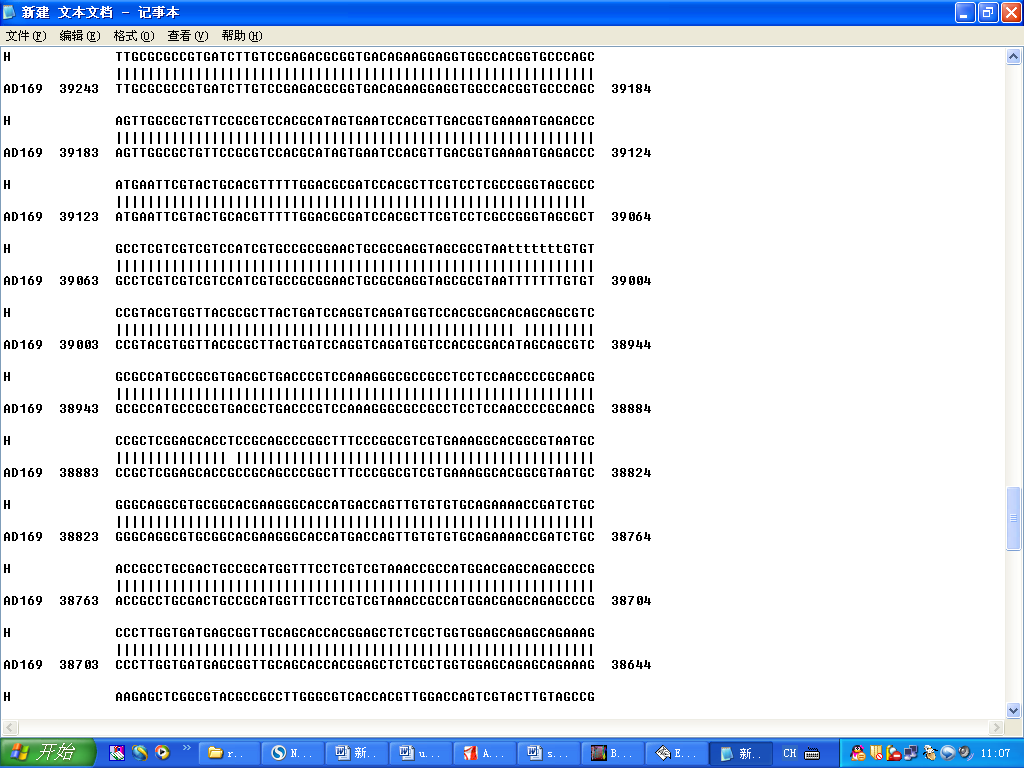


**CREB/CEBP, E2F,, nt38998-38986**

**5’end, nt38946**

**UL31anti-2 ORF, nt 38938**


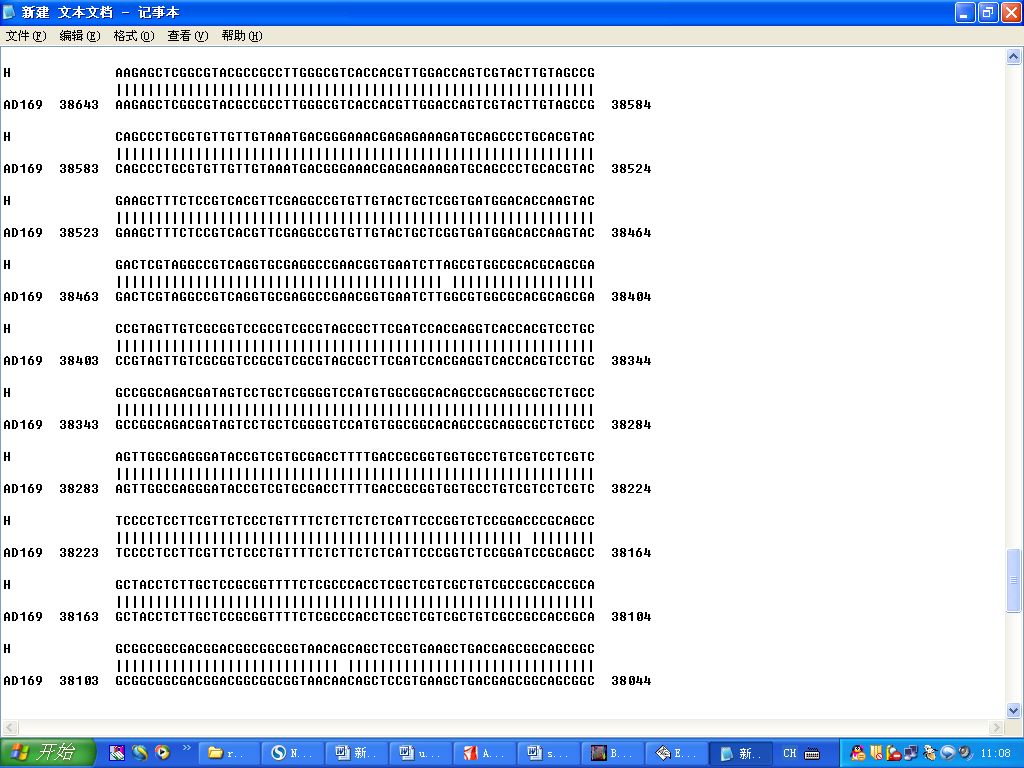


**TATA, nt38565-38559**

**5’end, nt38566**

**UL31anti-2 ORF, nt 38377**

**UL31anti-3 ORF, nt 38313**


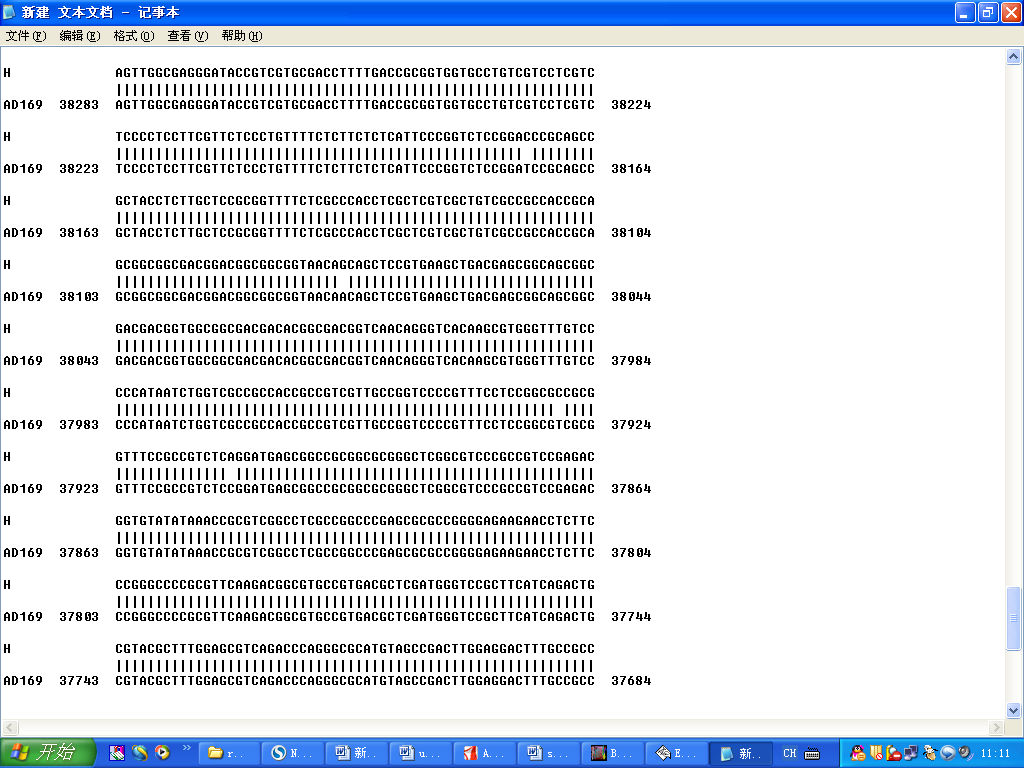


**GATA, nt38276-38269**

**5’end, nt38264**

**UL31anti-3 ORF, nt 37903**

**TATA, nt37865-31859**

**5’end, nt37836**

**5’end, nt37824**


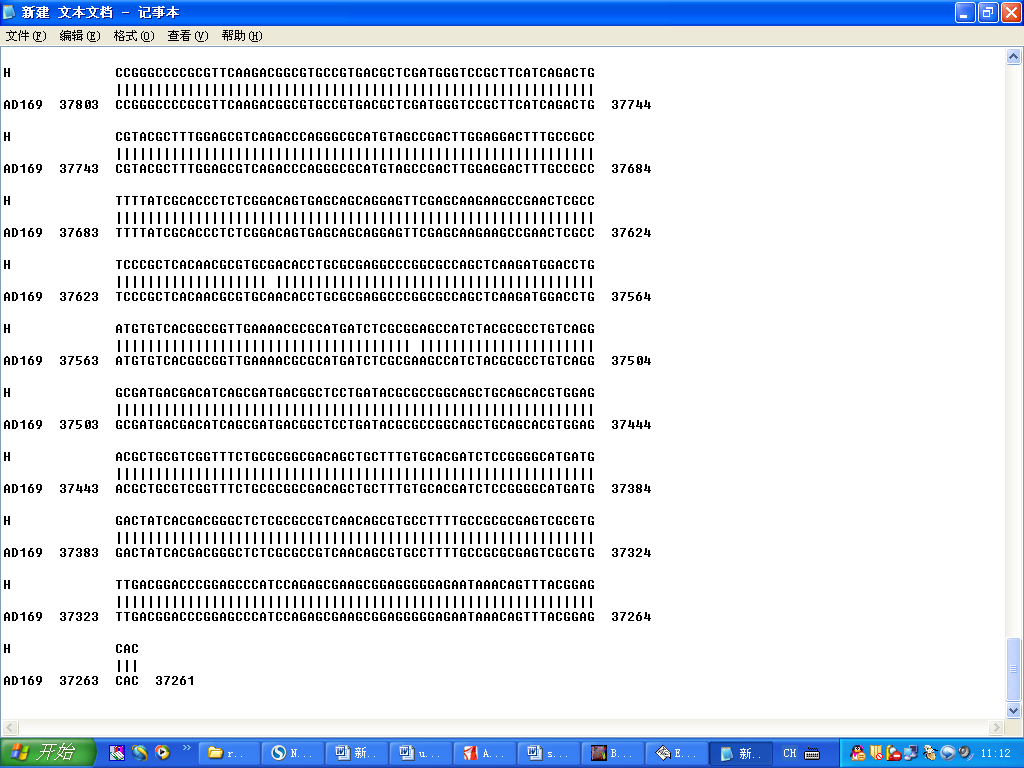


**PolyA, 37281-37276**

**Supplement Fig**. Alignment of UL30-UL32 DNA sequences of HCMV H strain and AD169 strain. Relative positions are shown as AD169 strain (GenBank: X17403.1). The predicted ORFs are denoted by square brackets. The 5′ends of the transcripts are remarked by triangles. The regulate elements are remarked by barres.
